# Ontario adults’ health behaviors, mental health, and overall well-being during the COVID-19 pandemic

**DOI:** 10.1186/s12889-021-11732-6

**Published:** 2021-09-15

**Authors:** Katie J. Shillington, Leigh M. Vanderloo, Shauna M. Burke, Victor Ng, Patricia Tucker, Jennifer D. Irwin

**Affiliations:** 1 Health and Rehabilitation Sciences Program, Faculty of Health Sciences, University of Western Ontario, London, Ontario Canada; 2 School of Occupational Therapy, Faculty of Health Sciences, University of Western Ontario, London, Ontario Canada; 3 School of Health Studies, Faculty of Health Sciences, University of Western Ontario, London, Ontario Canada; 4 Schulich School of Medicine and Dentistry, University of Western Ontario, London, Ontario Canada; 5 Department of Family and Community Medicine, University of Toronto, Toronto, Ontario Canada; 6 Division of Professional and Practice Support, College of Family Physicians of Canada, Mississauga, Ontario Canada

**Keywords:** Physical activity, Sedentary behavior, Diet, Well-being, Mental health, COVID-19

## Abstract

**Background:**

Public health measures such as physical distancing and work-from-home initiatives have been implemented to slow the spread of COVID-19. These measures may also be associated with unhealthy lifestyle behaviors, which could be particularly problematic for those already at highest risk for losing years of healthy life due to chronic disease (i.e., 30–59-year-olds). The purpose of this paper is two-fold: (1) to provide an overview of Ontario adults’ health behaviors (i.e., physical activity, sedentary behaviors, and dietary intake), mental health, and well-being during the first few months of the COVID-19 pandemic (April–July 2020); and (2) to explore the difference between physical activity and various health behaviors (i.e., well-being, mental health, and dietary intake).

**Methods:**

As a part of a larger, longitudinal study, participants completed an online survey that included demographic information, the Global Physical Activity Questionnaire, Starting the Conversation, the Mental Health Inventory, and the Personal Wellbeing Index-Adult. Data analyses involved computing measures of central tendency and dispersion for demographic characteristics and tools followed by descriptive statistics. Separate independent *t*-tests were conducted to investigate the difference between physical activity status and well-being, mental health, and dietary intake.

**Results:**

A total of 2157 Ontarians completed an online survey. Descriptive statistics indicated that respondents met physical activity and sedentary behavior guidelines, reported double the amount of recommended recreational screen time, practiced moderately healthy dietary behaviors, experienced mental health problems, and scored below “normal” in some well-being domains.

**Conclusion:**

As the end of the COVID-19 pandemic is currently unknown, its associated restrictions and society changes may influence adults’ behaviors in both the short- and longer-term. As such, our findings might provide immediate insight into the development of timely and evidence-informed health promotion and disease prevention strategies for Canadians, which could support adults’ health behaviors, mental health, and well-being during the COVID-19 pandemic and other, future pandemics.

## Introduction

COVID-19 was declared a global pandemic by the World Health Organization (WHO) in March 2020. In Canada, a state of emergency was declared between March 12–22, 2020 (province and territory dependant) [1]. Travel restrictions, the physical closure of schools and universities, and closing of many businesses resulted [1]. Changes to everyday activities and routines (e.g., physical distancing, intense personal hygiene practices, working from home) have been necessitated by public health mandates, and these changes have impacted leisure and work practices for many citizens [2]. These changes might also be associated with wide-spread impacts on adults’ health. In fact, researchers recently reported that, because of similar restrictions mandated in other countries, adults have experienced a decline in health behaviors, well-being, and mental health [3–6]. Many of the outcomes noted above are associated with increased risk of chronic disease and therefore, impacts to them may be particularly concerning for those already at highest risk for losing years of healthy life due to chronic disease (i.e., disability adjusted life years; adults aged 30–59) [7–9]. Variations in COVID-19 rates and subsequent public health measures across Canada and around the world necessitate the need to explore these behaviors at a local level. The current paper is intended to compliment and fill in current gaps in understanding the health behaviors (i.e., physical activity, sedentary behaviors, and dietary intake), mental health, and well-being of adults during the early months of the COVID-19 pandemic.

The COVID-19 pandemic continues to impact the health of Canadian adults. Researchers of studies conducted during the same timeframe as the current study have found that the pandemic has influenced Canadians’ physical activity both positively [10] and negatively [2, 10, 11] while their screen time [12] and sedentary behavior have increased [11]. Canadian adults also reported higher levels of distress and negative mental health as a result of the pandemic [11, 13], as well as improvements in healthy food consumption [14] and increased junk food consumption [12] compared to pre-pandemic. Specifically, Di Sebastiano and colleagues [2] conducted a 10-week nation-wide study to investigate changes in the physical activity levels of Canadian adults (*n* = 2338, 35–44 years) prior to and immediately following the introduction of physical distancing guidelines in Canada. Participants were sampled using a physical activity tracking app that collected the physical activity data of app users [2]. The authors found that levels of objectively measured moderate-to-vigorous physical activity (MVPA), light physical activity (LPA), and steps per day (measured via a national physical activity tracking app) significantly decreased as a result of the pandemic [2]. Similarly, Peterson and colleagues [10] explored how the pandemic impacted the physical activity and perceptions of health among adults in Calgary, a Canadian city, using a grounded theory methodology. A maximum variation sampling strategy was utilized to recruit 12 adults (20–70 years) during the months of June to October 2020 [10]. The authors concluded that the COVID-19 pandemic impacted the daily routines of participants (e.g., work, school, home, family life, socializing) and the pandemic had both positive and negative effects on participants’ physical activity and perceptions of health [10]. Jenkins and colleagues [13] administered a cross-sectional survey to Canadian adults (*n* = 3000; aged 18+) with the goal of investigating the impact of the pandemic on their mental health. The researchers distributed the survey via a national polling vendor who deployed the survey to a random selection of Canadian adults, stratified by Canadian Census-informed socioeconomic characteristics [13]. The authors concluded that Canadian populations were experiencing a deterioration in mental health and coping strategies as a result of the pandemic [13]. Lamarche and colleagues [14] investigated the change in diet habits and quality of adults (*n* = 2495; aged 18+ years) during the early stages of the COVID-19 pandemic lockdown in Quebec, Canada. The researchers recruited participants via a multimedia campaign, based on a needs assessment, and administered questionnaires before (June 2019–February 2020) and during (April–May 2020) early lockdown [14]. Interestingly, they found that diet quality improved slightly, from pre- to during lockdown [14]. In contrast, Zajacova and colleagues [12] assessed changes in Canadian adults’ (*n* = 4383; aged 25+) health behaviors (e.g., junk food consumption and screen time) during the early stages of the COVID-19 pandemic. To inform their work, the authors used publically available data from the Canadian Perspectives Survey Series 1: Impacts of COVID-19 (CPSS-COVID), a cross-sectional survey administered by Statistics Canada [12]. The researchers found that 25% of participants increased their junk food consumption and 60% of participants increased their screen time during the early stages of the pandemic (March–April 2020) [12]. Similarly, Woodruff and colleagues [11] explored how the stress, physical activity, and sedentary behaviors of Canadian adults (*n* = 121; aged 18+) changed during the early stages (April–May 2020) of the COVID-19 pandemic. The researchers recruited participants via social media advertisements; participants were asked to complete a fillable calendar with their step counts and and answer an online survey [11]. They found that participants’ sedentary behavior and stress (daily and work-related) increased, while their physical activity decreased as a result of the COVID-19 pandemic [11].

The COVID-19 pandemic has not only influenced the lives of Canadian adults but has also negatively impacted adults on a global scale. Most notably, and similar to what has been found in Canadian studies, adults’ physical activity has decreased [4–6, 15], their sedentary behavior has increased [5, 6, 15], and they are reporting higher levels of distress and negative mental health [5, 16], as well as weight gain and unhealthy food consumption [15, 17]. Specifically, Zheng and colleagues [6] conducted a study to investigate the physical activity, sedentary behavior, and sleep of young adults (*n* = 631, 18–35 years) during the initial stages of the COVID-19 pandemic in China (April 15–26, 2020). Participants were recruited via online advertisements and word of mouth and were sent a survey administered through Google forms [6]. The researchers concluded that there was an inverse relationship between physical activity and sedentary behavior, such that participants’ physical activity levels declined significantly with concurrent increases in their sedentary time [6]. In the United States, Meyer and colleagues [5] evaluated the impact of the pandemic (April 3–8, 2020) on adults’ levels of physical activity, sedentary behavior, and mental health (*n* = 3052, 18–24 years). Both convenience and snowball sampling were used to recruit participants [5]. Self-report data was collected cross-sectionally, wherein participants reflected on pre- and post-COVID health behaviors [5]. These authors also concluded that there was a decline in participants’ physical activity levels and an increase in their sedentary behavior which, in turn, were associated with higher negative mental health and lower positive mental health [5]. This was found to be particularly true for those who were previously active, as well as those who had self-isolated/quarantined [5]. Lopez-Bueno and colleagues [4] investigated the physical activity levels of adults in Spain during mandated confinement (March 22–29, 2020), via a cross-sectional survey. Individuals were recruited through social media and convenience sampling was used to select study participants [4]. The researchers found that participants’ weekly physical activity levels declined by 20% (i.e., approximately 45 min of physical activity per week) [4]. Researchers administered an international online survey to examine how COVID-19 home confinement (April 2020) impacted adults’ levels of physical activity and sedentary time, as well as their nutrition behaviors [15]. Participants were recruited via email, social media platforms, and faculty websites and were administered a survey, that was reviewed and edited by over 50 researchers worldwide, through Google forms [15]. Ammar and colleagues [15] surveyed adults (*n* = 1047, aged 18+) primarily from Asia, Africa, and Europe and concluded that home confinement had a negative impact on all physical activity intensity levels, and participants’ daily sitting time increased from 5 to 8 h. Further, participants reported engaging in increased unhealthy food consumption and meal patterns during confinement [15]. In Poland, Sidor and Rzymski [17] administered an online survey to adults (*n* = 1097, aged 18+ years) to investigate nutritional and consumer habits during the nationwide quarantine period (April 17–May 1, 2020). This survey was self-designed and not based on previously validated scales [17]. The authors concluded that 43% of participants reported eating more and 52% reported snacking more during quarantine, and that these behaviors were more common in individuals with overweight and obesity [17]. Further, nearly 30% of respondents reported weight gain and an increased BMI that was associated with low vegetable, fruit, and legume consumption, as well as high consumption of meat, dairy, and fast-food [17]. Mazza and colleagues’ [16] investigation of Italian adults (*n* = 2766) revealed psychological distress during COVID-19 (May 18–22, 2020). The authors administered a cross-sectional online survey and concluded that, compared to European epidemiological statistics, participants demonstrated high and very high levels of distress [16]. The researchers also found a significant association between being female and increased depression, anxiety, and stress [16]. In a study conducted in the United Kingdom by White and Van Der Boor [18], the authors investigated the impact of the COVID-19 pandemic – inclusive of the initial lockdown period (March 31–April 13, 2020) – on the mental health and well-being of adults. A convenience sample of participants were recruited via social media platforms and a cross-sectional online survey was administered [18]. Participants that self-isolated prior to the lockdown reported increased feelings of isolation, and the majority reported poorer mental health, well-being, and quality of life leading from concerns about their livelihood due to COVID-19 [18].

Worth noting are the methodology strategies utilized in the above-described studies. Specifically, in the Canadian studies sampling methods ranged from maximum variation sampling [10] to random sampling stratified by census information [13]. Other Canadian studies did not recruit participants but rather analyzed publicly available population data [12] or utilized data available from physical activity tracking apps [2]. Studies conducted outside of Canada primarily utilized convenience [4, 5, 18] and snowball sampling [4]. While the majority of the studies (both Canadian and external) were cross-sectional and survey-based, they differed in terms of rigour. For example, Meyer and colleagues [5] relied on retrospective self-report data, a method of data collection where participants tend to overestimate their responses and demonstrate recall bias [19, 20]. To combat retrospective data collection and recall bias, researchers use technology such as wearable activity trackers [11] and apps [2]. Lamarche and colleagues [14] improved the rigour of their recruitment and data collection process through use of a needs assessment and Ammar and colleagues [15] administered a survey that was reviewed by 50 experts in the field prior to dissemination. In contrast, some researchers created surveys without the inclusion of valid measurements [17], and others strictly used publicly available data [12]. The use of previously validated and reliable instruments in surveys has been recognized as crucial in social and health science research [21]. The decision to create a survey without valid and reliable measurements alters the integrity of the tool, which is concerning. Further, using publicly available data has posed ethical concerns, as described in a recent analysis conducted by Stommel and de Rijk [22].

It is evident, based on the literature reviewed above, that the COVID-19 pandemic has, on a global scale, negatively influenced individuals’ health behaviors, mental health, and well-being. The impact of the pandemic on the full complement of these outcomes among the various provinces of Canada remains unclear, as none of the studies described above have investigated these outcomes strictly in the province of Ontario. Despite Canada’s federated model of government, each province is responsible for organizing their own health systems with variations based on population needs. Consequently, each province has not experienced COVID-19 in the same ways, inclusive of prevalence rates and provincially mandated public health measures. Further, to our knowledge no studies conducted in Ontario have explored the difference between physical activity and well-being, mental health, and dietary intake, respectively. Given the work conducted by Meyers and colleagues [5] in the United States – who found that participants’ physical activity levels were negatively correlated with their sedentary behavior and mental health – there is a need to also explore this within Canadian populations. Additionally, one study described above used publicly available population data instead of recruiting participants [12], which warrants caution as secondary data collection is at a greater risk for biases and error compared to primary data collection [23]. Another study [17] used tools that were suitable for responding to the study purpose but were not validated, thus requiring caution when interpreting the findings. As such, there is a need for studies with primary data collection and valid and reliable measurements. To this end, the purpose of this paper is two-fold: (1) to provide an overview of Ontario adults’ health behaviors (i.e., physical activity, sedentary behaviors, and dietary intake), mental health, and well-being during the first few months of the COVID-19 pandemic (April–July 2020); and (2) to explore the difference between physical activity and various health behaviors (i.e., well-being, mental health, and dietary intake).

## Methods

The current paper is a part of the Health Outcomes for adults during and following the COVID-19 PandEmic (HOPE) longitudinal study, which aims to assess the impact of COVID-19 restrictions on the lifestyle-related health behaviors and overall wellbeing of Ontario adults during and following the pandemic. The current paper uses baseline data from the larger study, collected between April 24 and July 13, 2020. The study received ethics approval from Western University’s Health Sciences Research Ethics Board (HSREB #115827).

### Study procedures

Participants were recruited via social media advertisements (i.e., Facebook, Twitter, LinkedIn, and Instagram). In addition, regional health units, community health centres, and medical clinics were invited to circulate the advertisement. To be included in the study, participants needed to be: 1. an Ontario resident; 2. between the ages of 30–59 years at baseline; and 3. able to read and write in English. A power calculation deemed that a sample size of 244 was sufficient to achieve 80% power at a significance level of 0.05.

### Measures

The tools used were selected based on their validity, brevity, and suitability for the study’s target population. All tools required self-report and were administered online via Qualtrics as one survey. To diminish social desirability bias, honesty demands [24] were employed at the beginning of the survey. That is, at the beginning of the survey the following instructions were provided for participants: ‘We ask you to please answer the following questions as honestly as possible. There are no right or wrong answers to any of the questions. Whatever you truly think or feel is the answer you should pick.’

#### Demographic questionnaire

The demographic questionnaire included 14 items such as age, sex, gender, ethnicity, and highest level of education achieved.

#### Health behaviors

##### Global physical activity questionnaire (GPAQ)

The GPAQ was previously validated for use among adults and measures physical activity at the population level [25]. The GPAQ includes four domains: 1. activity at work; 2. travel to and from places; 3. recreational activities; and 4. sedentary behavior. For the purpose of this study, the recreational activities (6 items) and sedentary behavior (1 item) components of the GPAQ were measured only, given the restrictions in place that prevented many citizens from traveling anywhere and necessitating many working from home [i.e., given the local mandated restrictions, the nature of individuals’ work might have changed, resultantly confounding responses to scale questions (e.g., ‘In a typical week, on how many days do you do vigorous-intensity activities as part of your work?)]. Examples of recreational activities and sedentary behavior questions included: ‘How much time do you spend doing moderate-intensity sports, fitness or recreational (leisure) activities on a typical (pandemic) day?’; and ‘How much time do you usually spend sitting or reclining on a typical day?’. A second question was added to the sedentary behavior component regarding screen time, as screen time is encompassed within Canada’s newly released 24-Hour Movement Guidelines [26]. This question was not included in the overall sedentary behavior domain, but rather it was analyzed separately.

##### Starting the conversation (STC)

The STC was previously validated for use among adults and is used to identify individuals’ dietary patterns [27]. The authors did not report traditionally used validity data; however, the STC items and summary score were moderately intercorrelated (*r* = 0.39–0.59, *p* < 0.05). It includes 8 items that ask individuals to identify the frequency with which they engaged in certain dietary behaviors over the past month from a set of response categories (e.g., less than 1 time (0), 1–3 times (1), 4 or more times (2)). Examples of questions include: ‘How many times a week did you eat fast food meals or snacks?’ and ‘How many servings of vegetables did you eat each day?’

#### Mental health

##### Mental health inventory (MHI-5)

The MHI-5 has been previously validated (AUC = 0.892) [28] for use among adults and measures mental health status using 5 items: 2 regarding general positive affect and 1 for each anxiety, depression, and behavioral/emotional control. Participants were asked how much time over the past month they felt each statement to be true of them on a 6-point scale ranging from ‘all of the time’ (1) to ‘none of the time’ (6). Examples of questions include: ‘How much of the time during the last month have you felt downhearted and blue?’ and ‘How much of the time during the last month have you been a very nervous person?’

#### Well-being

##### Personal well-being index-adult (PWI-A)

The PWI-A was previously validated (Cronbach’s α range from 0.70 and 0.85) [29] and includes 7 items corresponding to quality of life domains (i.e., standard of living, health, achieving in life, relationships, safety, community-connectedness, and future security) and 2 optional items (i.e., satisfaction with life as a whole, and spirituality or religion) [29]. Participants were asked to indicate how satisfied they felt in each of the domains on a scale from 0 to 10 (0 being no satisfaction at all and 10 being completely satisfied). Examples of questions included: ‘How satisfied are you with your personal relationships?’ and ‘How satisfied are you with what you are achieving in life?’. The question ‘How satisfied are you with your health?’ was altered to more specifically ask participants how satisfied they were with their 1. mental health and 2. physical health.

### Data analysis

All analyses were completed in SPSS (version 26). Data analyses involved computing measures of central tendency and dispersion for demographic characteristics and tools followed by descriptive statistics.

#### Health behaviors

##### Global physical activity questionnaire (GPAQ)

The scoring protocol, published by WHO [30], which recommends calculating total time and percentage of time for each domain, was used to identify participants’: total recreational related physical activity in minutes per week and average total recreational activity in minutes per day (i.e., setting specific physical activity); the percentage of participants classified as doing no recreational-related physical activity; the total time spent in sedentary activities per day; the total recreational moderate-intensity minutes per week; and the total recreational vigorous intensity minutes per week. Total minutes per week spent engaged in moderate-to-vigorous intensity physical activity could also be calculated.

##### Starting the conversation (STC)

To score the STC, all items were summed to yield a total score on a scale ranging from 0 to 16, with lower scores reflecting a healthier diet and higher scores indicating a need for improvement [27].

#### Mental health

##### Mental health inventory (MHI-5)

To score the MHI-5 [28], items 3 (‘Have you felt calm and peaceful?’) and 6 (‘Have you been a happy person?’) were reverse coded and then the scores for each item were summed. The raw scores were then transformed to a 0–100-point scale, where a score of 100 represents optimal mental health.

#### Well-being

##### Personal well-being index-adult (PWI-A)

To score the PWI-A scale [29], all data (including the optional items) were converted to the standard 0–100 scale format (e.g., a score of 7 becomes 70 points). The PWI-A is scored by analyzing each domain as a separate variable or by summing the scores to yield an average that represents ‘subjective well-being’. We analysed the items separately, to account for the change made to the satisfaction with health question (i.e., the question ‘How satisfied are you with your health?’ was divided into two questions to ask participants how satisfied they were with their mental health and physical health). Per the scoring protocol [29], participants who consistently indicated a maximum [10] or minimum (0) score on all domains were removed prior to analysis because such data may indicate lack of understanding. Higher scores indicate better well-being and the normative range for means of Western samples is 70–80 [29]. For a detailed account of the scoring protocol refer to the International Wellbeing Group [29].

#### Difference between physical activity status and various health outcomes

Separate independent *t*-tests were conducted to investigate the difference between physical activity status (no engagement in moderate-to-vigorous physical activity vs. engagement in moderate-to-vigorous physical activity) and various health outcomes including well-being (satisfaction with life as a whole), mental health, and dietary intake. A Bonferroni correction was used to adjust for multiple comparison bias and type 1 error inflation (α/3 = 0.017).

## Results

### Demographics

The survey was completed by 2157 participants with a mean age of 43.2 years (*SD* = 8.8). Those who reported that they were suspected to receive a formal diagnosis of COVID-19 (*n* = 32) were excluded from the analyses; due to the small *n*-size, it was not possible to conduct separate sub-analyses to compare across groups. Of the included participants, the majority identified as female (*n* = 1718; 89.4%) and Caucasian (*n* = 1760; 91.5%). Most reported that they were married/common law/engaged (*n* = 1508; 78.1%) and having completed a university undergraduate degree (*n* = 543; 28.1%) or higher (*n* = 563; 29.1%). The majority of participants reported being employed full-time (*n* = 1147; 59.4%), with an average income between $80,000–$110,999 (*n* = 371; 19.2%) and $111,000–$150,000 (*n* = 382; 19.8%). For a comprehensive overview of demographic characteristics, see Table 1.

**Table 1:** Baseline Demographic Information of Ontario Adults During the Initial Stages of the COVID-19 Pandemic (April–July 2020)

Participant Characteristics (*n* = 1931)	*n*	%
Age, *M* (*SD*)		
	43.20 (8.8)	
Sex		
Male	199	10.4
Female	1718	89.4
Gender		
Male	197	10.3
Female	1713	89.5
Non-binary	1	0.1
Ethnicity		
Indigenous	20	1.0
Caucasian (White)	1760	91.5
South Asian	38	2.0
Chinese	21	1.1
Black	9	0.5
Filipino	5	0.3
Latin American	14	0.7
Arab	4	0.2
Southeast Asian	5	0.3
Korean	3	0.2
Japanese	4	0.2
Multiracial	19	1.0
Metis	3	0.1
Marital Status		
Single	238	12.3
Married/common law/engaged	1508	78.1
Divorced/separated	155	8.0
Widowed	18	0.9
Highest Education Achieved		
Less than high school	23	1.2
High school completed	148	7.7
Community college and/or journeyman apprenticeship completed	606	31.4
University undergraduate degree completed	543	28.1
University graduate degree or higher completed	563	29.1
Other	42	2.2
Employment Status		
Employed full-time	1147	59.4
Employed part-time	153	7.9
Unemployed	199	10.3
Casual	33	1.7
Other	388	20.1
Income		
< $30,000	94	4.9
$30,000 - $59,999	234	12.1
$60,000 - $79,999	220	11.4
$80,000 - $110,999	371	19.2
$111,000 - $150,000	382	19.8
> $150,000	449	23.3

*Note.* The total sample size was 2157 participants; not all categories summed to equal the total sample due to missing data. Age was collected as a continuous variable

### Health behaviors

The mean score for total recreational physical activity in minutes per week was 297.7 (5.0 h; *SD* = 415.8), while a total of 43% of participants were classified as doing no recreational-related physical activity. Individuals reported spending an average of 426.2 min (7.1 h; *SD* = 244.8) per day sitting or reclining (not including time spent sleeping) and an average of 359.4 min (6.0 h; *SD* = 207.0) per day on screens. The mean score for the total time spent engaging in moderate-intensity physical activity in minutes per week was 199.4 (3.3 h; *SD* = 272.4) and the mean score for the total time spent engaging in vigorous-intensity physical activity in minutes per week was 97.3 (1.6 h; *SD* = 225.7). In terms of dietary assessment, the score for the STC was 7.1 (*SD* = 2.6). The scores of individual items can be found in Table 2.

**Table 2:** Ontario Adults’ Health Behaviors During the Initial Stages of the COVID-19 Pandemic (April–July 2020)

Scale	Total *N*	Mean (*SD*)	Range	Frequency *n* (%)	
*Global Physical Activity Questionnaire (GPAQ)*					
Total recreational PA in min/wk	2006	297.7 (415.8)	0–5460		
Percentage of participants classified as doing no recreational-related PA	2031	0.43	0–1.00		
Total minutes spent sitting or reclining in a typical day	2001	426.2 (244.8)	0–1380		
Total minutes spent on screens in a typical day	2005	359.4 (207.0)	0–1320		
Total moderate-intensity min/wk	2010	199.4 (272.4)	0–2730		
Total vigorous-intensity min/wk	2042	97.3 (225.7)	0–3360		
*Starting the Conversation (STC; Dietary assessment)*					
Over the past few months …					
how many times a week did you eat fast food meals or snacks?	1947			< 1 time/wk	901 (46.3)
				1–3 times/wk	774 (39.7)
				4+ times/wk	272 (14.0)
how many servings of fruit did you eat each day?	1943			5+ times/day	85 (4.4)
				3–4 times/day	590 (30.3)
				< 3 times/day	1268 (65.3)
how many servings of vegetables did you eat each day?	1946			5+ times/day	240 (12.3)
				3–4 times/day	892 (45.8)
				< 3 times/day	814 (41.8)
how many regular sodas/pop or glasses of sweet tea did you drink each day?	1946			< 1 time/day	1449 (74.5)
				1–2 times/day	365 (18.7)
				3+ times/day	132 (6.8)
how many times a week did you eat beans (like pinto or black beans), chicken or fish?	1944			3+ times/wk	1147 (59.0)
				1–2 times/wk	577 (29.7)
				< 1 times/wk	220 (11.3)
how many times a week did you eat regular snack chips or crackers (not low fat)?	1947			< 2 times	716 (36.8)
				2–3 times	819 (42.1)
				4+ times	412 (21.1)
how many times a week did you eat desserts and other sweets (not the low fat kind)?	1947			< 2 times/wk	537 (27.6)
				2–3 times/wk	737 (37.9)
				4+ times/wk	673 (34.5)
how much butter or margarine (or meat fat) do you use to season or put on vegetables, potatoes, or bread?	1947			Very little	701 (36.0)
				Some	976 (50.1)
				A lot	270 (13.9)
Total score	1936	7.12 (2.6)	0–15	0–5	533 (27.5)
				6–10	2161 (62.8)
				11–15	188 (9.7)

*Note.* Missing participants ranged from 5.3–7.2% for the GPAQ and 9.7–10.2% for the STC

### Mental health

The mean mental health score using data from the MHI-5 was 60.30 (*SD* = 19.1). The scores of individual items can be found in Table 3.

**Table 3:** Ontario Adults’ Mental Health During the Initial Stages of the COVID-19 Pandemic (April–July 2020)

Scale	Total *N*	Mean (*SD*)	Range	Frequency *n* (%)	
*Mental Health Inventory (MHI)*					
How much of the time during the past month have you …					
been a very nervous person?	2118			All of the time	57 (2.7)
				Most of the time	214 (10.1)
				A good bit of the time	399 (18.8)
				Some of the time	614 (29.0)
				A little bit of the time	676 (31.9)
				None of the time	158 (7.5)
felt so down in the dumps that nothing could cheer you up?	2117			All of the time	12 (0.6)
				Most of the time	86 (4.1)
				A good bit of the time	236 (11.1)
				Some of the time	445 (21.0)
				A little bit of the time	719 (34.0)
				None of the time	619 (29.2)
felt calm and peaceful?	2119			All of the time	18 (0.8)
				Most of the time	394 (18.6)
				A good bit of the time	546 (25.8)
				Some of the time	593 (28.0)
				A little bit of the time	484 (22.8)
				None of the time	84 (4.0)
felt downhearted and blue?	2118			All of the time	17 (0.8)
				Most of the time	139 (6.6)
				A good bit of the time	305 (14.4)
				Some of the time	570 (26.9)
				A little bit of the time	871 (41.1)
				None of the time	216 (10.2)
been a happy person?	2119			All of the time	26 (1.2)
				Most of the time	611 (28.8)
				A good bit of the time	568 (26.8)
				Some of the time	566 (26.7)
				A little bit of the time	313 (14.8)
				None of the time	35 (1.7)
Total score	2117	60.30 (19.1)	0–100	0–20	70 (3.2)
				21–40	331 (17.3)
				41–60	632 (34.5)
				61–80	819 (38.8)
				81–100	265 (12.5)

*Note*. Missing participants ranged from 1.8–1.9%

### Well-being

The mean response to each domain of the PWI-A across all participants satisfaction with: life as a whole was 69.0 (*SD* = 19.2); their standard of living was 76.8 (*SD* = 18.9); and their health, which was further broken down by the researchers to include physical and mental health, were 64.6 (*SD* = 20.7) and 64.4 (*SD* = 21.9), respectively. The mean score for participants’ satisfaction with: what they were achieving in life was 68.9 (*SD* = 20.7); their personal relationships was 72.0 (*SD* = 21.2); their safety was 75.6 (*SD* = 20.4); their satisfaction with feeling part of their communities was 64.1 (*SD* = 23.2); their future security was 64.6 (*SD* = 22.4); and their spirituality/religion was 73.3 (*SD* = 25.2). The scores of individual items can be found in Table 4.

**Table 4:** Ontario Adults’ Well-Being During the Initial Stages of the COVID-19 Pandemic (April–July 2020)

Scale	Total *N*	Mean (*SD*)	Range
*Personal Well-Being Index-Adult (PWI-A)*			
How satisfied are you with …			
your life as whole?	2150	69.0 (19.2)	0–100
your standard of living?	2151	76.8 (18.9)	
your physical health?	2150	64.6 (20.7)	
your mental health?	2150	64.4 (21.9)	
what you are achieving in life?	2150	68.9 (20.7)	
your personal relationships?	2149	72.0 (21.2)	
how safe you feel?	2150	75.6 (20.4)	
feeling part of your community?	2151	64.1 (23.2)	
your future security?	2148	64.6 (22.4)	
your spirituality or religion?	2137	73.3 (25.2)	

*Note*. Missing participants ranged from 0.0–0.7%

### Difference between physical activity status and various health outcomes

Results from the independent sample *t*-tests indicated evidence of a difference between participants’ physical activity status and their well-being (*t* (2024) = − 5.47, *p* = < 0.001, 95% CI: − 7.13 to − 3.36; Table 5), mental health (*t* (2027) = − 6.50, *p* = < 0.001, 95% CI: − 8.10 to − 4.34; Table 6), and dietary intake (*t* (1923) = 10.86, *p* = < 0.001, 95% CI: 1.18 to 1.69; Table 7) based on physical activity status.

**Table 5:** Difference Between Physical Activity Status and Wellbeing Among Ontario Adults During the Initial Stages of the COVID-19 Pandemic (April–July 2020)

Group	*n*	Mean	*SD*	*df*	*t*-value	*p*	Lower 95% CI	Upper 95% CI
No MVPA	530	65.30	20.61	2024	−5.47	< 0.001	−7.13	−3.36
MVPA	1496	70.55	18.36	844.63	−5.18	< 0.001	−7.23	−3.26

*Note*. CI = confidence interval; MVPA = moderate-to-vigorous physical activity. Significance was set at 0.05

**Table 6:** Difference Between Physical Activity Status and Mental Health Among Ontario Adults During the Initial Stages of the COVID-19 Pandemic (April–July 2020)

Group	*n*	Mean	*SD*	*df*	*t*-value	*p*	Lower 95% CI	Upper 95% CI
No MVPA	532	55.79	20.10	2027	−6.50	< 0.001	−8.10	−4.34
MVPA	1497	62.01	18.54	872.64	−6.25	< 0.001	−8.17	−4.27

*Note*. CI = confidence interval; MVPA = moderate-to-vigorous physical activity. Significance was set at 0.05

**Table 7:** Difference Between Physical Activity Status and Dietary Intake Among Ontario Adults During the Initial Stages of the COVID-19 Pandemic (April–July 2020)

Group	*n*	Mean	*SD*	*df*	*t*-value	*p*	Lower 95% CI	Upper 95% CI
No MVPA	496	8.18	2.67	1923	10.86	< 0.001	1.18	1.69
MVPA	1429	6.74	2.49	812.66	10.49	< 0.001	1.17	1.70

*Note*. CI = confidence interval; MVPA = moderate-to-vigorous physical activity. Significance was set at 0.05

## Discussion

The purpose of this paper was to provide an overview of the health behaviors (physical activity, sedentary behavior, and dietary intake), mental health, and well-being of adults in Ontario during the first few months of the COVID-19 pandemic (April–July 2020). The findings underscore the importance of focusing on healthy behaviors to support positive mental health and well-being during the COVID-19 pandemic and will be discussed below.

With respondents self-reporting 199 min of moderate physical activity and 97 min of vigorous physical activity per week, our sample, on average, met the physical activity goal identified in the newly released Canadian 24-Hour Movement Guidelines for Adults, which recommend at least 150 min of MVPA per week as well as several hours of LPA [26]. This finding aligns with the qualitative work of Peterson and colleagues [10]; participants in their study described how the COVID-19 pandemic positively influenced their physical activity, as many participants adapted and developed strategies to maintain their pre-pandemic fitness levels. Similarly, with respondents indicating 7 h per day engaged in sedentary pursuits, our sample also, on average, fell below the recommended threshold of 8 h or less according to the guidelines. Interestingly, this finding differs from previous Canadian research conducted by Woodruff and colleagues [11], who found that sedentary behavior increased during the early months of the pandemic. The difference in findings may be attributed to the fact that Woodruff and colleagues [11] included participants across Canada, though the majority of their sample also resided in Ontario. More likely to explain the difference, the authors measured physical activity using daily step count via a wearable activity tracker [11]. It is known that individuals tend to over-estimate their levels of physical activity when using self-report measures [31] and thus, it is likely that the work by Woodruff and colleagues [11] is a more accurate reflection of the physical activity levels in Canada. However, with respect to screen time, respondents reported about double the amount of recommended recreational use (at 6 h per day versus the guideline of no more than 3). That said, recreational- and work-related screen use were not distinct variables within the tool and as such, it is plausible that a portion of the reported screen use was for reasons other than recreation. Our findings are in line with those by Lesser and Nienhuis [32], who conducted a nationally representative study to investigate the impact of COVID-19 on Canadian adults’ (*n* = 1098) levels of physical activity and well-being. They found that 33% of individuals who were classified as “inactive” became more active and 40.3% of individuals classified as “active” also became more active during the months of April and early May 2020 (i.e., during the initial public health mandates in Canada) [32]. This may be due to a surge in participants engagement in home-based exercise, which can have both physical and psychological benefits [33]. However, it is worth noting that approximately 43% of participants in the current study were classified as engaging in *no* recreational-related physical activity, which is concerning given that the data was collected during the spring/summer, a time when individuals are typically more active than in the winter months [34]. It is plausible that this number might increase as the pandemic continues into the winter months and environments become colder. It is also worth noting that participants who engaged in MVPA reported significant improvements in their wellbeing and mental health and consumed a healthier diet than those who did not engage in MVPA. This is not surprising given the plethora of evidence to support the positive association between physical activity and numerous health outcomes [35–37]. Such trends are important to consider and observe over time, given the longitudinal nature of the current study.

The average score for participants’ dietary intake (i.e., 7.12 on a scale that ranged from 0 to 15) suggests that participants reported eating moderately healthy [27]. In a pre-pandemic Canadian survey, 28.6% of individuals (12+ years) reported consuming fruits and vegetables five or more times per day [38]. By contrast, in the current study, approximately 4% and just over 11% of participants reported consuming fruits and vegetables five or more times per day, respectively. In a pre-pandemic study conducted by Nardocci and colleagues [39], high processed foods were found to have made up nearly half (45%) of the daily calories consumed by Canadian adults and were positively associated with obesity. In the current study, more than one third of participants reported eating fast food/snacks 1–3 times/week, and 14% reported this for 4 or more times per week. Similarly, in a Canadian study conducted by Zajacova and colleagues [12], the authors found that 25% of participants increased their junk food consumption during the early stages of the COVID-19 pandemic. These numbers are alarming as consumption of high processed food, such as some fast food, are highly correlated with the development of chronic disease (e.g., obesity, diabetes, cancer) [40]. In other recent studies investigating adults’ dietary habits during COVID-19 confinement/lockdown periods globally, researchers have also reported increased unhealthy food consumption [15], low fruit and vegetable consumption, and high consumption of fast food [17].

The average score for participants’ mental health (i.e., 60.3) was somewhat concerning. For interpretation, researchers have typically chosen MHI-5 cut scores ranging from 70 to 76 to identify mental health problems [41–43]. Therefore, it appears that many participants may have experienced mental health problems and challenges during the early stages of the pandemic. This is consistent with previous research conducted in Canada during the COVID-19 pandemic, as researchers found that participants are experiencing a deterioration in mental health and coping strategies as a result of the pandemic [13]. While there could be many reasons for participants’ poor mental health, based on previous research, it is possible that these findings could, in part, be associated with the dramatic changes/restrictions citizens experienced during Ontario’s most stringent public health mandates. For instance, although fewer people were impacted directly, the Torontonians who were quarantined during the severe acute respiratory syndrome (SARS) outbreak in 2003 experienced substantial psychological distress and depression [44]. Regardless of their causes, our findings are consistent with a systematic review conducted by Xiong and colleagues [45], who found that symptoms of anxiety, depression, post-traumatic stress disorder, psychological distress, and stress during the COVID-19 pandemic were reported by individuals in China, Spain, Italy, Iran, the US, Turkey, Nepal, and Denmark. Similarly, in a secondary analysis of a national, longitudinal cohort study conducted by Pierce and colleagues [46] (*n* = 17,452) the authors found that the mental distress of adults’ (aged 16+) increased by roughly 8% one month into lockdown (April 23–30, 2020) in the United Kingdom (UK). In another UK-based study, O’Connor and colleagues [47] surveyed 3044 adults (aged 18+) during the first month of lockdown (March 31–April 9, 2020) and found that suicidal ideation increased over time. Interestingly, the authors found that symptoms of anxiety decreased, and depressive symptoms and feelings of loneliness did not change [46, 47]. The discrepancy in findings between the two UK-based studies may be due to the difference in sample size and timeframe of data collection, as Pierce and colleagues [46] sampled a larger population further into the COVID-19 pandemic. Thus, while O’Connor and colleagues [47] did not see significant changes in participants’ mental health this may be because their sample size was smaller and they collected data early into the COVID-19 pandemic, when perhaps participants had not experienced the effects of the pandemic to the fullest extent.

Per the tool’s scoring protocol, participants’ well-being was below the “normative” range (i.e., 70–80 points) for means in Western populations in several domains, as measured via the PWI-A [29]. Specifically, participants scored about 5–6 points below the low end of “normal” when asked how satisfied they were with their physical and mental health, respectively. Equally concerning were participants’ scores regarding their satisfaction with feeling part of their communities and their future security, as they also had average scores that were more than 5 points below “normal”. Our findings suggest that, on average, participants experienced a rather poor sense of well-being in these domains during the first few months of the pandemic in Ontario. That said, regarding their satisfaction with life as a whole and what they are achieving in life, participants were within decimals of falling into the “normal” range, with average scores of 69.0 and 68.9, respectively. Worth noting are the domains that participants scored within the range deemed “normal”, including their satisfaction with their standard of living, their personal relationships, their safety, and their spirituality/religion. Interestingly, participants scores were within the “normative” range regarding their satisfaction with safety, but below the “normative” range in terms of their anticipated future security (e.g., financial or job security). It is possible that one such reason for this might be due to individuals’ fear of potential repercussions of the pandemic, which could negatively influence their future security. Additionally, it was suspected that individuals’ scores would be below “normal” in terms of their satisfaction with personal relationships and spirituality/religion, given that people might have experienced feelings of isolation/loneliness due to limited physical contact and as a result of places of worship being closed due to public health restrictions [48], respectively; however, this was not the case. It is possible that participants connected with others virtually, rather than in-person, thus maintaining their personal relationships [48, 49]. Further, 78.1% of the sample identified as being married/common law/engaged, which might also explain our findings. Many places of worship also offered virtual services, providing individuals with the opportunity to practice their spirituality/religion [49, 50].

### Strengths, limitations, and future directions

There are several strengths to this study. First, to the best of our knowledge, this is the first study to provide an overview of Ontario adults’ well-being, mental health, physical activity, sedentary behavior, and dietary intake during the early months of the COVID-19 pandemic. The sample was large (> 2000) and the tools used were all previously validated while being sufficiently brief to minimize participant burden and increase completion rates. Nevertheless, there are also limitations worth noting. First, all data were collected using self-report measures which have the tendency to lend themselves to social desirability bias. However, given the size of the sample, nature of the pandemic, and the government restrictions in place, it was not possible to collect data via wearables and as such, this limitation was unavoidable. Honesty demands were employed to limit the risk of bias [24]. Second, while participants’ screen time use was measured, it was determined via only one question. We were unable to locate a brief previously validated tool to assess screen use, and as such, one question was used to collect these data. As a result, we did not specify recreational versus work-related screen use and were unable to compare our results to the recommended guidelines. Lastly, the demographics of our sample limit the generalizability of our study. Most of our sample identified as white females of high socioeconomic status, having completed an undergraduate degree or higher. Given that the sample of participants is fairly well-educated and higher income, they might not face barriers to being physical activity, compared to those with lower education and incomes. Further, the high proportion of females in the current study might be attributed to our recruitment methods. Participants were recruited via social media platforms (i.e., Facebook, Twitter, Instagram, and LinkedIn), which women reportedly use more than men [51]. Future studies might utilize stratified sampling and include an exploration of the impact of the pandemic on the lifestyle-related behaviors, mental health, and well-being of multiple genders, less affluent individuals, and other ethnicities.

## Conclusion

A new “normal” has emerged because of the COVID-19 pandemic – one that includes physical distancing, wearing masks, and restrictions on social gatherings [52, 53]. During the strictest public health mandates to date, Ontario adults self-reported below average well-being, mental health challenges, moderately healthy dietary behaviors, and appeared to meet physical activity and sedentary behavior guidelines. Findings from the current paper may aid in the preparedness for subsequent iterations of strict, pandemic-related public health mandates. Our findings might be immediately useful to encourage the development of timely and evidence-informed health promotion and disease prevention strategies for Ontarians. This could include the development of physical activity interventions and mental health resources to help citizens navigate their lives in as healthy ways as possible during future pandemics or future waves of the current pandemic. Our findings might also provide insights about Ontario women aged 30–59, as researchers have concluded that women have been disproportionately impacted by the pandemic compared to other genders [12, 54]. Strategies such as these could support adults’ health behaviors, mental health, and well-being during the COVID-19 pandemic and other, future pandemics.

## Data Availability

The datasets used and/or analysed during the current study are available from the corresponding author on reasonable request.
